# Editorial: Unlocking potential: cognitive rehabilitation for individuals with acquired brain injuries

**DOI:** 10.3389/fnhum.2025.1730344

**Published:** 2025-11-21

**Authors:** Asha K. Vas, Gail Poskey, Martin Rice, Michelle Scheffler, Lori Cook

**Affiliations:** 1 School of Occupational Therapy, Texas Woman's University, Dallas, TX, United States; 2 Department of Neurosurgery, Houston Methodist Research Institute, Houston, TX, United States; 3 Center for BrainHealth^®^, School of Behavioral and Brain Sciences, The University of Texas at Dallas, Dallas, TX, United States

**Keywords:** assessment, brain injury, cognition, cognitive rehabilitation, daily function, technology

Acquired brain injuries (ABI), including traumatic brain injury (TBI), stroke, and other neurological insults, remain a leading cause of long-term disability worldwide. Survivors often face persistent challenges that span cognition, emotion, physical functioning, and social participation, with ripple effects on families and communities. Cognitive rehabilitation has emerged as a cornerstone of recovery, offering strategies to improve attention, memory, executive function, and self-regulation. Yet, critical questions remain regarding the mechanisms that support recovery, the effectiveness of various approaches, and how best to translate gains into real-world outcomes. This Research Topic brings together several contributions that collectively expand the evidence base, highlight innovative interventions, and address methodological gaps in cognitive rehabilitation science. Together, they underscore the need for rehabilitation strategies that are neuroscience-driven, individualized, and attuned to the complexities of daily life. The Research Topic has already been a tremendous success, garnering more than 8,000 views and downloads to date, underscoring its resonance and impact in the field.

## Technology-enhanced rehabilitation

Technology offers unprecedented opportunities to expand access and tailor cognitive interventions. One study introduced a guided experiential skill learning system for individuals with chronic TBI. By embedding state regulation skills (SRS) practice into digital goal-based tasks, and integrating therapist-led guidance, the intervention promoted goal-directed functioning even at a distance. Results from a small cohort suggest that remote, technology-augmented rehabilitation can bridge critical gaps in service delivery, a finding with profound relevance in resource-limited or rural contexts.

In a complementary randomized controlled trial, researchers evaluated computer-assisted cognitive training (CACT) for post-stroke cognitive impairment. Patients receiving CACT alongside conventional therapy showed greater improvements in executive function, memory, and activities of daily living than controls. The study illustrates how targeted digital programs can amplify standard rehabilitation, supporting a blended model of care that leverages both technology and therapist expertise.

Complementing this, a network meta-analysis of cognitive training interventions for post-stroke cognitive impairment synthesized evidence from fifty randomized controlled trials. Results indicated that combining traditional and computer-based training yields superior improvements in cognition, daily functioning, and motor outcomes compared to single modalities. Virtual reality and exercise-based approaches also showed promise, reinforcing the value of multimodal interventions. By ranking the relative effectiveness of available programs, this analysis provides clinicians with actionable guidance for tailoring interventions to patient needs.

## Physical- cognitive interplay

Researchers increasingly acknowledge the relationship between physical capacity and cognition. A population-based study using NHANES data demonstrated a positive correlation between handgrip strength (HGS) and cognitive performance in individuals with head injury. Gender differences emerged, with men showing stronger associations with memory and processing speed, and women with global cognition and verbal fluency. These findings suggest that simple physical measures may serve as proxies for cognitive status and highlight the need to integrate physical and cognitive rehabilitation for holistic recovery.

## Efficacy of cognitive training protocols

Efficiency and scalability are key considerations in cognitive rehabilitation. A large RCT compared Strategic Memory Advanced Reasoning Training (SMART) with the Study of Cognitive Rehabilitation Effectiveness (SCORE) in active-duty service members with mild TBI. Both groups improved in higher-order cognition, but SMART achieved comparable outcomes in 60% fewer treatment hours. Importantly, benefits were consistent regardless of comorbid PTSD severity, emphasizing the robustness of training-based gains even in complex clinical populations. Extending this line of work, a pilot study applied SMART to young women with stroke, a population often underrepresented in rehabilitation research. Participants showed gains in executive function, cognitive self-efficacy, and stress reduction, with strong feasibility ratings. These results highlight both the adaptability of SMART and the importance of designing interventions responsive to gender- and age-specific needs.

## Functional outcomes in daily life

Cognitive rehabilitation must support everyday functioning. A multicenter study examined driving ability in individuals with non-progressive ABI, focusing on the utility of neuropsychological testing and on-road assessments in licensure decisions. The results are expected to inform regulatory practices in France, where new policies mandate multiprofessional evaluation but lack consensus on appropriate assessment tools. By linking clinical practice with public policy, this research addresses autonomy, safety, and quality of life at the societal level.

Similarly, a Norwegian study explored the relationship between cognitive performance and health-related quality of life 4 years post-stroke. Memory, reaction time, and fine motor coordination emerged as key predictors of cognitive-social-mental outcomes, underscoring the long-term interplay between cognitive abilities and perceived wellbeing. These findings reinforce the need for rehabilitation strategies that extend beyond the acute phase and continue to address cognitive and psychosocial domains years after injury.

## Psychometric advances

Robust measurement tools are essential to advance rehabilitation science. A multicenter project introduced the Fondazione Don Gnocchi Clinical Complication Scale (FDG-CCS), developed specifically for patients with severe ABI. The scale demonstrated good inter-rater reliability and validity against established measures, capturing complications that often derail recovery. Its adoption could improve monitoring, enhance clinical decision-making, and optimize rehabilitation planning in complex cases.

## Conclusion

Collectively, the studies in this Research Topic affirm that cognitive rehabilitation is both a science and an art, grounded in neuroscience and aligned with the daily function of individuals with ABI. The articles highlight persistent challenges, including the need for long-term follow-up and scalable models of care. Together, they call for strategies that are effective, personalized, and sustainable, restoring both cognitive function and independence. Looking ahead, these contributions inspire innovation, collaboration, and translation into practice, with the future of cognitive rehabilitation driven by advancing functional cognition, integrating innovative technologies, and empowering individuals with ABI to realize their potential and fully participate in life.

